# Health Disparities and Converging Epidemics in Jail Populations: Protocol for a Mixed-Methods Study

**DOI:** 10.2196/10337

**Published:** 2018-10-24

**Authors:** Robert T Trotter II, Ricky Camplain, Emery R Eaves, Viacheslav Y Fofanov, Natalia O Dmitrieva, Crystal M Hepp, Meghan Warren, Brianna A Barrios, Nicole Pagel, Alyssa Mayer, Julie A Baldwin

**Affiliations:** 1 Center for Health Equity Research Northern Arizona University Flagstaff, AZ United States; 2 Department of Anthropology Northern Arizona University Flagstaff, AZ United States; 3 School of Informatics, Computing, and Cyber Systems Northern Arizona University Flagstaff, AZ United States; 4 Department of Psychological Sciences Northern Arizona University Flagstaff, AZ United States; 5 Department of Physical Therapy and Athletic Training Northern Arizona University Flagstaff, AZ United States; 6 Department of Nursing and Health Professions University of South Carolina Beaufort, SC United States; 7 Department of Health Sciences Northern Arizona University Flagstaff, AZ United States

**Keywords:** behavioral health, chronic illness, health status, health care utilization, incarceration, infectious disease, jail, recidivism

## Abstract

**Background:**

Incarcerated populations have increased in the last 20 years and >12 million individuals cycle in and out of jails each year. Previous research has predominately focused on the prison population. However, a substantial gap exists in understanding the health, well-being, and health care utilization patterns in jail populations.

**Objective:**

This pilot study has 5 main objectives: (1) define recidivists of the jail system, individuals characterized by high incarceration rates; (2) describe and compare the demographic and clinical characteristics of incarcerated individuals; (3) identify jail-associated health disparities; (4) estimate associations between incarceration and health; and (5) describe model patterns in health care and jail utilization.

**Methods:**

The project has two processes—a secondary data analysis and primary data collection—which includes a cross-sectional health survey and biological sample collection to investigate infectious disease characteristics of the jail population. This protocol contains pilot elements in four areas: (1) instrument validity and reliability; (2) individual item assessment; (3) proof of concept of content and database accessibility; and (4) pilot test of the “honest broker” system. Secondary data analysis includes the analysis of 6 distinct databases, each covered by a formal memorandum of agreement between Northern Arizona University and the designated institution: (1) the Superior Court of Arizona Public Case Finder database; (2) North Country Health Care; (3) Health Choice Integrated Care; (4) Criminal Justice Information Services; (5) Correctional Electronic Medical Records; and (6) iLEADS. We will perform data integration processes using an automated honest broker design. We will administer a cross-sectional health survey, which includes questions about health status, health history, health care utilization, substance use practices, physical activity, adverse childhood events, and behavioral health, among 200 Coconino County Detention Facility inmates. Concurrent with the survey administration, we will collect Methicillin-resistant and Methicillin-sensitive *Staphylococcus aureus* (samples from the nose) and dental microbiome (*Streptococcus sobrinus* and *Streptococcus mutans* samples from the mouth) from consenting participants.

**Results:**

To date, we have permission to link data across acquired databases. We have initiated data transfer, protection, and initial assessment of the 6 secondary databases. Of 199 inmates consented and enrolled, we have permission from 97.0% (193/199) to access and link electronic medical and incarceration records to their survey responses, and 95.0% (189/199) of interviewed inmates have given nasal and buccal swabs for analysis of S. aureus and the dental microbiome.

**Conclusions:**

This study is designed to increase the understanding of health needs and health care utilization patterns among jail populations, with a special emphasis on frequently incarcerated individuals. Our findings will help identify intervention points throughout the criminal justice and health care systems to improve health and reduce health disparities among jail inmates.

**International Registered Report Identifier (IRRID):**

RR1-10.2196/10337

## Introduction

County jail systems, throughout the United States, are more of a broken public health system, and less of an effective justice system than anyone assumes. That condition should be effectively changed on both directions, criminal and public health.

Criminal Justice Coordinating Council director

In the last 2 decades, jail and prison populations of the United States have increased markedly in size and diversity [1,2], with a concomitant impact on public health. More than 20 million Americans are currently or have been previously incarcerated, with 12 million cycling in and out of jails each year [3,4]. In addition, there are significant nationwide racial and ethnic disparities in the criminal justice system; 60% of jail and prison populations are ethnic and racial minorities, although they make up just 30% of the general US population [1,3,5]. Prison and jail minority populations are disproportionately burdened by higher rates of substance abuse and poor mental health, as well as chronic and communicable diseases [3,6-8], with considerable rates of comorbidity [8] creating complex prevention and treatment conditions for these institutions. Compared with the general population, prison inmates have a higher burden of mental and neurological disorders, have high levels of stress, anxiety, sleep deprivation, and depression and have lower levels of self-efficacy as a result of the stigma and loss of social ties associated with being incarcerated [7-12]. Rates of many chronic diseases in US jails and prisons are more than double of those in the general population—diabetes (5.0% vs 2.4%), chronic respiratory conditions (eg, chronic obstructive pulmonary disease, 34.1% vs 19.2%), and liver disease (10% vs 0.6%) [3]. Similarly, the rates of communicable diseases, such as hepatitis C, HIV, and tuberculosis [5,13], are higher in incarcerated populations (eg, 3.5% vs 0.4% for HIV among 25-34-year olds) [14]. Women [15], ethnic minorities [16], and older adults [17] are considered particularly at-risk for poor health outcomes in the jail system. Furthermore, people who do not have a permanent residence in between jail stays face greater risk of mortality because of treatable conditions [18].

Previous research on incarceration and health has predominately focused on relatively stable prison populations, in spite of the potentially greater impact of jail incarceration on public health and population health conditions. In contrast to prison populations, jail populations are considerably more transient and more likely to interact regularly with the general population. The average length of jail stays is 8-10 days, with a small percentage of the populations staying for up to 1 year or longer awaiting trial [19,20]. The shorter-term stay and release from jail can be a destabilizing public health force for individuals and communities, making them more likely to relapse to substance abuse and nonadherence to mental and physical health treatment programs [21]. In addition, they become an equally important potential vector for a communicable disease being cycled in and out of jail populations. Continuity of care is severely impacted by cycling between jail and community and is associated with a limited opportunity for stability in health care [22]. By federal regulation, Medicaid benefits, which provide insurance for a disproportionate number of inmates prior to their incarceration, are suspended or even eliminated upon incarceration [22,23]; this creates a barrier to continuity of care for many chronic conditions, treatment regimens for severe mental impairments (SMI), and other behavioral health problems, because of benefits being temporarily or permanently terminated, as well as marked differences in the formularies offered by the jail as opposed to those offered by Medicaid benefits.

Nationwide, high health care utilization among criminal justice system populations has been observed both before and after incarceration. The risk of hospitalization is higher following release from correctional facilities compared with the general population [24]. Over 80% of recently released jail and prison inmates have chronic mental, behavioral, and substance abuse issues, and 85% of these individuals rely on the emergency department to address acute episodes rather than seeking primary care from a physician [25-27]. Similarly, >50% of recently released jail inmates reported using emergency departments as their primary source of health care in the 3 months before jail incarceration, and 75% reported using acute care services at any time before being incarcerated [17]. Furthermore, to the best of our knowledge, no study has assessed the impact of the implementation of the Affordable Care Act and the Affordable Care Act’s Medicaid expansion on health care utilization among incarcerated individuals.

**Diseases of interest.**

**Infectious diseases**

- Hepatitis C
- HIV/AIDS
- Gonorrhea
- Syphilis
- Chlamydia
- Tuberculosis
- Methicillin-resistant *Staphylococcus aureus*, Methicillin-sensitive *Staphylococcus aureus*

**Cardiovascular diseases**

- Ischemic heart disease
- Heart failure
- Acute myocardial infarction
- Stroke or transient ischemic attack
- Atrial fibrillation
- Anemia

**Pulmonary diseases**

- Chronic obstructive pulmonary disease
- Asthma

**Cancer**

- Colorectal
- Endometrial
- Breast (female and male)
- Lung
- Prostate
- Leukemia and lymphomas

**Other chronic diseases or conditions**

- Chronic back pain (lumbago)
- Chronic kidney disease
- Diabetes
- Hypertension
- Hyperlipidemia
- Liver disease, cirrhosis, and other liver conditions (except viral hepatitis)
- Migraine and chronic headache

**Mental disorders and behavioral health**

- Depression
- Depressive disorders
- Bipolar disorder
- Anxiety disorders
- Alcohol use disorders
- Drug use disorders
- Autism spectrum disorders
- Intellectual disabilities and related conditions
- Learning disabilities
- Schizophrenia
- Posttraumatic stress disorder

**Table 1:** The demographic information of Coconino County compared with Arizona and the United States, 2016.

Demographic information		Coconino County (N=138,064)	Arizona (N=6,728,577)	United States (N=318,558,162)
**Race, n (%)**				
	Non-Hispanic white	75,521 (54.7)	3,734,360 (55.5)	195,276,153 (61.3)
	Black	1,933 (1.4)	3,297,000 (4.9)	42,368,236 (13.3)
	Native American or Alaska Native	37,968 (27.5)	363,343 (5.4)	4,141,256 (1.3)
	Hispanic	19,053 (13.8)	2,079,130 (30.9)	56,703,353(17.8)
	Other	3,590 (2.6)	222,043 (3.3)	20,069,164 (6.3)
Language other than English spoken at home, n (%)		33,550 (24.3)	1,809,987 (26.9)	66,897,214 (21.0)
High school graduate, n (%)		122,049 (88.4)	5,786,576 (86.0)	276,189,926 (86.7)
Median household income, US $		50,234	50,255	53,889
Persons in poverty, n (%)		26,922 (19.5)	1,103,487 (16.4)	40,456,887 (12.7)
Population per square mile		7.2	56.3	87.4
Uninsured (<65 years), n (%)		20,157 (14.6)	8,000,7001 (11.9)	32,174,374 (10.1)

Surveillance of both chronic and acute illness, intervention, and coordination of support services has the potential to improve health within the jail system [28,29], interrupt cycles of recidivism [30], and provide necessary continuity and support for transitions from jail to the community [27]. In addition to surveillance and targeted intervention programs, effective intervention points are needed to disrupt the cycle of homelessness and recidivism and address the comorbid occurrence of substance abuse and mental health issues [21,31,32]. Northern Arizona, specifically Coconino County, is particularly well-suited for studying the confluence and flow between the criminal justice and health care systems.

Unlike large metropolitan areas with multiple different hospital systems and emergency departments, Coconino County is a relatively isolated population with a single major health care system, single incarceration unit, and limited variability in public health resources.

Both secondary data analyses of linked criminal justice and health care system databases in Northern Arizona and the prospective health disparities survey of inmates, described below, are anticipated to provide information on racial and ethnic health disparities associated with a variety of chronic diseases, communicable diseases, and behavioral health issues. We have identified several diseases of interest (Textbox 1) and will determine the local rates of these targeted diseases and associated health disparities in Northern Arizona using the International Classification Diseases, Ninth and Tenth Revision Clinical Modification codes. Locally focused disparity research is essential in gaining an understanding of the population dynamics driving regional health care burdens. The population of Northern Arizona has a unique composition in that the number of American Indians is much higher compared with the United States as a whole (27.5% vs 1.3%), and Arizona ranks 10^th^ in the country for our uninsured population (Table 1) [33]. Higher representation of American Indians will provide sufficient statistical power to examine the needs of this underserved population.

The protocol described in this study is directed at understanding the longitudinal view of health care utilization before, during, and after jail incarceration. Results from assessing the complex trajectories of emergency department, behavioral health services (Regional Behavioral Health Authority), and public health services use (Department of Health) among individuals with high rates of recidivism, inmates characterized by a high number of jail admissions and discharges, will provide for targeted modeling of possible public health interventions for this population.

Evaluating and modeling the impact of individuals with high rates of recidivism on public health, using a “converging epidemics” or Syndemics Theory Approach [34], as opposed to a single condition epistemology, should allow us to identify multiple points for intervention, using a multisectoral rather than unintegrated approach to prevention and intervention. Understanding the comprehensive and interconnected needs, conditions, and health service utilization trajectories of the broken public health system in jails has significant potential to produce new models of intervention, effective policy change, and collective action.

## Methods

### Overview

The Health Disparities in Jail Populations project began, as demonstrated in the opening quote above, as a community-engaged research project with a strong interdisciplinary focus, including a partnership between Northern Arizona University’s (NAU) Center for Health Equity Research, School of Informatics, Computing, and Cyber Systems, College of Social and Behavioral Sciences, and the Coconino County Criminal Justice Coordinating Council (CJCC) [35]. The confluence of existing expertise in chronic disease, communicable disease, and behavioral health, as well as the social determinants of health among underserved populations, provides a unique opportunity for research and positive impact on our ability to intervene in population health issues and model potential areas of prevention and intervention in critically underinvestigated groups.

The operational questions framing the study, identified below, focus on the empirical definition of criminal justice recidivists, the social determinants of existing health disparities in a jail population compared with nonincarcerated individuals, the convergence of high-impact utilization patterns for health services in and out of jail, and identifying intervention points to address public health conditions for the incarcerated population.

We are guided by the Social Determinants of Health framework [36] to identify, assess, and develop our initial and evolving research questions on existing health disparities among jail populations. The Social Determinants of Health framework allows for exploration of the complex intersections of social-, cultural-, economic-, and system-level influences on health and well-being. The research questions framing this feasibility study are as follows:

### Research Questions

Our research questions are as follows:

1. Empirically define recidivists: What is the distribution of the number of incarcerations, lengths of stay, and other characteristics of multiple incarcerations for the population cycling through the Coconino County Detention Facility?
2. Describe and compare characteristics of incarcerated individuals: What are the demographics and socioeconomic characteristics of persons in the jail system?
3. Identify jail-associated health disparities: What are the incidence and prevalence rates for targeted diseases and conditions in the jail population and general health care populations in Northern Arizona? Are health disparity issues more similar to prison populations or the general population?
4. Describe associations between incarceration and health: Are incarceration variables (eg, length of stay, type of criminal activity, risk level, ability to post bail, and the number of incarcerations) associated with health outcomes and disease severity in jail populations?
5. Model patterns in health care and jail utilizations and identifying opportunities for intervention: What is the health care and jail utilization over the life span of incarcerated individuals and compared with the general population?
6. The project is functionally separated into two data collection and analysis processes that address the overarching questions above—(1) a comprehensive secondary data analysis component and (2) a primary data collection effort that includes a cross-sectional health survey component that incorporates self-reported information, collection of biological samples, and data from the available secondary databases.

### Secondary Data Analysis of the Coconino County Detention Facility Inmates

Our focus for the secondary data analysis will be county-level data derived from the 6 listed data sources. The primary focus for the analysis is the Coconino County Detention Facility in Flagstaff, Arizona. The Coconino County Detention Facility provides inmate housing for local, state, and federal law enforcement agencies and courts in Northern Arizona; it is a regional holding facility that houses both sentenced and unsentenced misdemeanor and felony offenders. The Flagstaff facility has an operating capacity of 477 beds (477/596, 80.0% of the total available beds is considered operating capacity because of inmate security classification requirements). Northern Arizona’s demographics differ from those of Arizona’s and the US population (Textbox 1); thus, results may not be generalizable to state and national populations but may be generalized to populations with a high proportion of minority residents and populations with high uninsured and poverty rates.

A subset of our analytical population will be individuals with high rates of recidivism in the jail system. We will conduct literature searches for established definitions of jail recidivism in public policy, political science, law, and public health literature, as well as conducting an empirical assessment of criminal justice databases on incarceration. We have created an analytical framework that allows us to determine the impact of multiple incarcerations on the health status of individuals. The incarceration data are available in both the secondary analysis datasets, and the datasets that are linked to the inmate survey. Because characteristics of inmates differ with varying lengths of stay (eg, a difference between inmates incarcerated for 1 day compared with inmates awaiting trial for over a year), when characterizing inmates and modeling inmates’ health care utilization, we will stratify our results by meaningful categories based on the length of stay. In addition, we will categorize inmates meaningfully by the length of stay and other incarceration characteristics when estimating associations between incarceration and health and identifying health disparities. To inform our decision on an empirical definition of recidivists, using criminal justice databases, described below, we will describe the characteristics and distributions of arrests, incarcerations, and recidivism among Coconino County Detention Facility inmates. Measures will include frequency of arrests, the frequency of incarcerations, total time incarcerated, and average length of stay in the Coconino County Detention Facility.

The secondary data analysis efforts are centered on descriptive analyses of 6 distinct databases from 5 participating partners:

1. The Superior Court of Arizona public database provides case information (Public Case Finder) for incarceration and court history from 177 of the 184 courts in Arizona and includes all Coconino County courts. The Case Finder database includes birthdate, case number, case title, offense category (traffic, noncriminal ordinance, criminal, etc), court filing date, judge, disposition date (and appearance status), citation description, closed date, and case activity.
2. Criminal Justice Information Services (CJIS) is a database provided by a division of the Federal Bureau of Investigation containing criminal history data. Each state has a repository for criminal history information that feeds into the CJIS division system. The Coconino County Adult Probation Department provided access to the Arizona Computerized Criminal History System, which contains all arrest and court results (eg, convictions and not guilty verdicts) for offenders within Arizona that get reported to the CJIS. The CJIS contains 23,340 individuals aged ≥18 years from January 1, 2010 to December 31, 2014.
3. iLEADS is the Coconino County Detention Facility’s inmate record system. The system automates the incarceration process from booking, screening, classification, and release. It is the primary system used by the jail to track information about an inmate. iLEADS contains 79,506 individuals aged ≥18 years from January 1, 2007 to May 31, 2018.
4. Correctional Electronic Medical Records is the Coconino County Detention Facility’s electronic medical records system. This database is used by medical staff within the jail to track medical history during incarceration.
5. North Country Health Care Electronic Health Records is the electronic medical record system utilized by the North Country Health Care System. North Country is the Federally Qualified Health Center for Northern Arizona and provides primary and oral health care to 14 communities in Northern Arizona. This database contains records from primary care visits, dental visits, obstetrician-gynecologist and pregnancy visits, laboratory results, and diagnoses that can be associated with incarceration records for Coconino County. North Country Health Care contains 117,301 nonduplicated individuals from January 1, 2010 to December 31, 2014.
6. The Health Choice Integrated Care is a collaboration between Health Choice, the managed care solutions division of IASIS Healthcare, and the Northern Arizona Regional Behavioral Health Authority, a managed care organization that has served the behavioral health needs of Northern Arizona residents for >50 years. Their electronic medical records database contains integrated health care (medical and behavioral) records for members with SMI and provides the primary behavioral and SMI records.

Several procedural steps are required to allow successful secondary data analysis of the databases, including (1) creating and testing a secure transfer and data storage process compliant with the Institutional Review Board (IRB), Health Insurance Portability and Accountability Act of 1996 (HIPAA), Criminal Justice, and other federal privacy and confidentiality regulations for data with personal health information and personal identifying information to the secure project server; (2) conversion of the databases to an appropriate analytical format (SAS, SPSS, etc); (3) exploratory data analysis of each database (ie, acquisition or creation of an appropriate data dictionary, review of the quality of the data, and including missing data); (4) assessment of the database for variables that are relevant to the research questions; (5) identification of all variables that can be used to link the datasets; and (6) data cleaning as necessary.

The databases described above will be integrated to provide a longitudinal view of an individual across multiple community-based health providers, criminal justice databases, and jail-based health services. We will perform data integration for the databases by using an honest broker design [36]. The automated honest broker process has the following three general steps: (1) creating a unique identifier for each individual present in each database; (2) identifying common individuals across databases, and establishment of a single identifier for each individual; and (3) propagating the individual identifier to the source databases, and the removal of all identifying information, save for the unique identifier, from the source databases. We have implemented and pilot-tested a version of the automated honest broker system to determine the feasibility of linking the datasets for the full (interagency and interinstitution) database integration. The honest broker algorithm is designed to look for and match records that represent the same entity or person across disparate databases; this algorithm generates matches by 3 variables—name (first and last), Social Security Number, and date of birth. The algorithm was tested first on linking 2 datasets, containing 40,000 and 250,000 unique individuals or “records,” respectively. The algorithm found 10,815 matches between both datasets. A third dataset was added with 105,000 individuals, and the algorithm found 7740 matches among all 3 datasets.

The advantage of this design is threefold: (1) it establishes a persistent key as the identifier, which can then be populated back to source systems to enable further analysis or interventions to be tracked across systems, providers, and time; (2) provides an enhanced level of assurance for privacy and adherence to IRB standards and protocols; and (3) separates analysis process and analysts from bias associated with individually identifiable data, while allowing the incorporation of individual-level covariates, predictors, and outcome variables to be visible.

### Cross-Sectional Health Survey Among Coconino County Detention Facility Inmates

We will conduct a cross-sectional health survey among 200 Coconino County Detention Facility inmates housed at the Coconino County Detention Facility. The survey includes questions about (1) basic demographic information of respondents, (2) respondents’ experience with the criminal justice system, (3) health care utilization patterns of respondents, (4) epidemiology of communicable disease, chronic illness, and behavioral health issues, and (5) health behaviors (eg, physical activity and smoking). The survey will take participants approximately 1 hour to complete.

Prior to any data collection for the jail project, investigators are required to complete the Prison Rape Elimination Act training and a volunteer safety training. Training included safety procedures and regulations within the Coconino County Detention Facility. In addition, investigators are under an obligation or a “duty to inform” when an inmate poses a threat to either themselves or others. Finally, to preserve confidentiality, audio monitoring and recording are suspended during survey sessions. However, there are special security considerations that include video monitoring.

Participants will be recruited from the Coconino County Detention Facility based on a stratified purposive, sampling strategy [37]. The detention center consists of 4 pods (F Pod, A Pod, B Pod, and C Pod) with a total of 21 dorms containing 28-32 beds per pod. The dorms are segmented by sex (male and female), by an internal risk assessment evaluation (low, medium, and high security), and by known conflicts among inmates. We will recruit between 12 and 15 individuals per dorm. We established a total recruitment target of 200 individuals (approximately 33.6% (200/596) of the total available beds, and 41.9% (200/477) of the operational census for the facility) to achieve a representative sample of the population.

The inclusion criteria for the survey includes (1) being currently incarcerated in the Coconino County Detention Center in Flagstaff, AZ; (2) being aged ≥18 years; (3) being able to read English; and (4) providing informed consent for participation. The exclusion criteria are (1) being aged <18 years of age; (2) having residence in a restricted dormitory in the jail; and (3) having a decision to not provide informed consent for participation. Individuals will be excluded if they are unable to consent because of cognitive impairment. The 4 restricted dormitories include dorms housing juveniles being charged as adults, an “administrative confinement” dorm, a dorm for individuals diagnosed with SMI that are not considered competent to consent to participation, and an administration dorm that houses protected individuals, such as former officers. There will be no exclusion on the basis of sex, ethnicity, or health status. Our study involves a vulnerable population—county jail inmates. Persons with SMI and homeless individuals will not be specifically targeted but will not be excluded. Owing to the high comorbidity of SMI and substance abuse in jail populations [38], we anticipate enrolling these individuals. Finally, pregnant women may be part of the jail population, and although not specifically targeted, pregnant women will not be excluded. The confidentiality of the vulnerable populations will be maintained just as for our entire population. We will store all research information on secure data servers by encrypting the data and the server as a whole, as well as by limiting access to all data to key research personnel only. No individuals will be identified or identifiable in reports or publications.

### Recruitment

A study team member and a jail staff member will describe the project to a dorm’s inmates. Interested participants who meet the eligibility requirements will be assigned a time to complete the survey in groups of 5. During a scheduled session, Coconino County Detention Facility personnel will escort inmates to a program room equipped with a one-way mirror, video (but not audio) recording, chairs and tables, and materials used for programs. Before beginning, a study team member will review the consent form with participants. The consent form includes 3 sections as follows: (1) study survey; (2) collection of biological samples (see Collection of Biological Samples among Coconino County Detention Facility Inmates below); and (3) permission to access jail medical records. If an individual does not consent to the study survey, they will be escorted back to their pod. Participants may continue if they do not consent to the collection of biological samples and access to jail medical records. Following consent, individuals will be given a second-generation iPad to complete the survey on the Qualtrics Office Survey Application (Provo, UT), a platform for administering surveys without an internet connection. If, at any given time, an individual no longer wishes to participate, they will be thanked for their participation and escorted back to their dorm, and any partial data will be erased from the iPad. For participants who consented to the biological sample collection, once an individual completes the survey, a team member will retrieve the iPad and begin collecting biological samples. Upon completion, participants will be thanked for their participation and will be provided with US $15 in commissary privileges or a gift card added to their personal belongings and accessed after release from jail. Participants will then be escorted back to their dorm. After completion of data collection, a jail nurse will provide Correctional Electronic Medical Records data to researchers for participants who consented to this aspect of the study.

### Instrument Development

The survey instrument is comprised of items and scales adapted from existing national health surveys of general populations [39], other measures of relevant health, and well-being constructs with high previously demonstrated validity and reliability [40], as well as instruments targeted at assessing incarcerated populations [41]. The final instrument includes questions relating to a broad list of health domains; specifically, a range of communicable diseases, commonly occurring chronic conditions, issues related to behavioral health and well-being, and other related constructs (eg, global self-rated health status), as well as a comprehensive set of questions assessing demographic and socioeconomic characteristics. The majority of the questions are taken from (1) the National Health Interview Survey [42], (2) the National Health and Nutrition Examination Survey [39], (3) the Behavioral Risk Factor Surveillance System [43], (4) Behavioral Risk Factor Surveillance System Adverse Childhood Experiences module [44], (5) Patient Health Questionnaire [45], (6) International Physical Activity Questionnaire [46], and (7) the National Inmate Survey [47]. In reviewing the candidate items for the instrument, we found that the majority of the health surveys exclude inmates from their sampling framework; most also do not identify the previous incarceration.

An initial instrument was assessed through a pilot cognitive debriefing process [48,49], to ensure (1) a consistent understanding of questions across participants, and (2) an alignment of participants’ understanding of items with the original intent of the questions. The interviews were administered to 4 undergraduate students attending NAU. Survey items that were problematic tended fall into the following 3 categories: (1) uncertainty about definitions of items or recognition of some diseases; (2) the request for additional questions; and (3) the desire for additional response options (eg, add “internet” as a response option to the question querying the type of place one goes to for health care). To keep the integrity of well-validated questions, we were unable to accommodate items of the third type. However, we were able to accommodate concerns of types 1 and 2 and, subsequently, constructed the final version of the instrument.

The final instrument was piloted with a volunteer group of 5 individuals incarcerated in the Coconino County Detention Center to see whether any significant issues were surrounding the use of the instruments in a jail population. The instrument validity and individual item comprehension were assessed through a small pilot test (5 inmates) and debriefing process. We wanted to determine whether there were any significant problems with reading level, item comprehension, the sensitivity of questions (especially alcohol and drug questions), and the range of time it took to complete the survey (between 25 and 50 minutes for this group). The pilot test indicated that even the slowest reader could complete the survey in the allotted time. Reading comprehension was generally acceptable; participants requested further clarification about 3 of the diseases listed, and the alcohol and drug questions were not considered sensitive. We will conduct a sensitivity analysis for all items and scales in the instrument, as this type of analysis has not been conducted for jail populations.

### Collection of Biological Samples Among Coconino County Detention Facility Inmates

The survey administration detailed above will be accompanied by biological sampling from the nose and mouth of volunteer participants. Nasal samples will be assayed for carriage of *Staphylococcus aureus* (both Methicillin-resistant and Methicillin-sensitive *S. aureus*), while oral samples will be evaluated for the presence of the known dental caries-causing bacteria, (*Streptococcus sobrinus* and *Streptococcus mutans* samples from the mouth). Each of these bacterium is known to be elevated nationwide in prison populations [50] and Northern Arizona’s general (nonincarcerated) population. The biological sample collection will allow us to build an important database on the interaction of 2 public health conditions that are of high salience for this population and potentially allow us to differentiate between the general detention population and recidivists.

To collect *S. aureus* samples from the nose, a participant will use a sterile, single-tipped swab flocked with soft nylon fiber (Fisher Scientific, catalog # 22-349-700) to gently rub the inside of both nostrils with the swab head for 1-2 seconds. The samples will be processed at NAU’s Pathogen and Microbiome Institute by a trained wet-lab technician. The technician will use standard aseptic techniques to inoculate (streak) a HardyCHROM *S. aureus* plate (Hardy Diagnostics, p/n G311). The swab will be stored, and should the original streaking process fail to yield bacterial colonies, the swabs will be used to restreak another plate. Our previous efforts (unpublished pilot data) indicate that ~30% of Northern Arizona residents are positive for *S. aureus*. As a result, we anticipate that ~30% of the samples (60 of 200) will be positive for the bacterium. We alternatively expect, based on increases in prison populations, that significantly >30% of samples will be positive.

To collect *S. sobrinus* and *S. mutans* samples from the mouth, a participant will use a sterile, single-tipped cotton swab to gently rub the swab along the gum line of the upper and lower jaw, spending approximately 1-2 seconds on each tooth. Similar to above, a trained wet-lab technician will use standard aseptic techniques to streak a partitioned HardyCHROM plate containing media for *S. sobrinus* on one side and *S. mutans* on the other side (Hardy Diagnostics). Based on the previous work, up to 40% of Northern Arizona residents have one or more decayed teeth, with an unknown fraction of those being carriers for *S. mutans* and *S. sobrinus* (data not published). As a result, we anticipate that up to 40% of our samples (80 of 200) will be positive for, at least, one of the caries-causing bacteria, specifically, preliminary data indicates 10-20 positives for *S. sobriunus*.

## Results

To date, we have established the feasibility in the acquisition of highly restricted databases, including permission to link data across those databases across multisectoral institutions that have not been previously linked. We have successfully completed memoranda of agreements for each database, allowing for “public health research” on the databases. We have accomplished the data acquisition, transfer, conversion, and variable assessment on the 6 secondary databases and are scheduled to complete that process for all databases within 60 days. In addition, we have demonstrated the feasibility of gaining access to the jail population and have IRB approval for both the secondary data analysis (NAU IRB approval: 934185) and the prospective data collection (NAU IRB approval: 1067490), as well as linking both prospective and secondary data sources for our survey population using the automated honest broker system.

## Discussion

The combined protocols for the 2 complementary approaches present a number of procedural and methodological challenges that are not often present in common jail or public health services frameworks. A primary challenge includes identifying appropriate existing databases that both singly and collectively allow us to address the following 5 interconnected research questions: (1) empirically defining the jail population segments in ways that allow us to compare and contrast the general population with recidivists; (2) describing and comparing characteristics of Coconino County Detention Facility inmates; (3) identifying incarceration-associated health disparities for communicable disease, chronic illness, mental health, and substance abuse in jail populations; (4) describing critical associations between incarceration information (such as number of incarcerations) and health and disease; and (5) linking individuals across these datasets, through the use of an honest broker system, in a way that allows us to understand the health services impact of recidivists. Although many databases were explored, databases not included were found not applicable to our study questions or not accessible.

The second primary challenge was acquiring ethically appropriate access and federally sanctioned protection for the databases. All databases are “owned” by public or semipublic institutions and require a “community-based” approach for data acquisition. In addition, each database contains personal identifying information and personal health information of inmates, a federally recognized vulnerable population. Federally recognized vulnerable populations require additional data safety protocols beyond the Common Rule and HIPAA compliance. Several databases were removed from consideration because of refusal by the owner. However, our assessment of the included databases suggests the existing data configuration is appropriate to achieve the overall goals and objectives of the project. Each database required the creation of a separate memorandum of understanding and a complex data-sharing protocol. Each memorandum of understanding includes a set of common data acquisition and data protection procedures that are (1) project-based, and (2) IRB- and HIPAA-approved.

Developing the health survey in the jail presented additional challenges, including the design and development of a valid data collection instrument. Candidate questions were drawn from a variety of nationally deployed surveys (see above for full listing). We discovered very few of the psychometrically validated health-related instruments have been adapted to prison or jail populations. As an example of the need for adaptation, questions on physical activity (International Physical Activity Questionnaire) include explanatory examples such as shoveling snow, riding a bicycle, taking a walk, etc; those examples do not effectively exemplify activities available in a jail exercise area. Similarly, questions regarding social interactions may be problematic because of limited opportunities to interact with family and friends outside of jail. Time-restricted questions pose similar problems. The average length of incarceration is 8-10 days [19,20]. Thus, if we are interested in, for example, the impact of incarceration with depressive symptoms measured by questions such as, “Over the past two weeks, have you been bothered by any of the following problems?,” Patient Health Questionaire-9 [45], we may not be able to distinguish the level of depression prior to incarceration from the level of depression during incarceration, as the 2-week period may include both conditions for inmates. To determine the appropriateness of our instrument, we will apply basic psychometric analyses and post hoc sensitivity analyses to examine the validity and reliability among jail inmates. This work will allow us to determine whether future studies should develop new instruments designed specifically for jail inmates or can appropriately modify existing questions adapted to jail incarceration.

One of our working assumptions is that jails may not be an appropriate venue to conduct prevention and intervention programs in all 4 areas of the converging epidemics in our study (chronic illness, behavioral health, communicable disease, and substance abuse) and that some multisectoral approaches are needed to address public health needs of this population comprehensively. At the same time, jails may play a pivotal role in addressing the overall impact of incarceration on public health. We believe a multisectoral or collective impact framework will be necessary to reduce the “broken public health” system in jails. As a consequence of these intersecting conditions, we aim to produce a model for prevention and intervention programs in all 4 areas of health, potentially resulting in a marked cost reduction for health care among inmates.

## Abbreviations

CJCC: Criminal Justice Coordinating Council
CJIS: Criminal Justice Information Services
HIPAA: Health Insurance Portability and Accountability Act of 1996
IRB: Institutional Review Board
NAU: Northern Arizona University
SMI: severe mental impairment
